# Mosquito Larvicidal Activity, Antimicrobial Activity, and Chemical Compositions of Essential Oils from Four Species of Myrtaceae from Central Vietnam

**DOI:** 10.3390/plants9040544

**Published:** 2020-04-22

**Authors:** Nguyen Thi Giang An, Le Thi Huong, Prabodh Satyal, Thieu Anh Tai, Do Ngoc Dai, Nguyen Huy Hung, Nguyen Thi Bich Ngoc, William N. Setzer

**Affiliations:** 1 School of Natural Science Education, Vinh University, 182 Le Duan, Vinh City 43000, Nghe An Province, Vietnam; nguyengianganbio@gmail.com (N.T.G.A.); lehuong223@gmail.com (L.T.H.); 2 Aromatic Plant Research Center, 230 N 1200 E, Suite 102, Lehi, UT 84043, USA; psatyal@aromaticplant.org; 3 Department of Pharmacy, Duy Tan University, 03 Quang Trung, Da Nang 50000, Vietnam; anhtai0808qn@gmail.com; 4 Graduate University of Science and Technology, Vietnam Academy of Science and Technology, 18-Hoang Quoc Viet, Cau Giay, Hanoi 10072, Vietnam; daidn23@gmail.com; 5 Faculty of Agriculture, Forestry and Fishery, Nghe An College of Economics, 51-Ly Tu Trong, Vinh City 4300, Nghe An Province, Vietnam; 6 Center for Advanced Chemistry, Institute of Research and Development, Duy Tan University, 03 Quang Trung, Da Nang 550000, Vietnam; 7 Pedagogical Institute of Science, Vinh University, 182 Le Duan, Vinh City 43000, Vietnam; ngockhoahoa@gmail.com; 8 Department of Chemistry, University of Alabama in Huntsville, Huntsville, AL 35899, USA

**Keywords:** *Baeckea frutescens*, *Callistemon citrinus*, *Melaleuca leucadendra*, *Syzygium nervosum*

## Abstract

Mosquitoes are important vectors of several diseases, and control of these insects is imperative for human health. Insecticides have proven useful in controlling mosquito populations, but insecticide resistance and environmental concerns are increasing. Additionally, emerging and re-emerging microbial infections are problematic. Essential oils have been shown to be promising mosquito larvicidal agents as well as antimicrobial agents. In this work, the essential oils from four species of Myrtaceae (*Baeckea frutescens*, *Callistemon citrinus*, *Melaleuca leucadendra*, and *Syzygium nervosum*) growing wild in central Vietnam have been obtained by hydrodistillation and analyzed by gas chromatographic techniques. The essential oils have been screened for mosquito larvicidal activity against *Aedes aegypti*, *Aedes albopictus*, and *Culex quinquefasciatus*, and for antimicrobial activity against *Enterococcus faecalis*, *Staphylococcus aureus*, and *Candida albicans*. *Callistemon citrinus* fruit essential oil, rich in α-pinene (35.1%), 1,8-cineole (32.4%), limonene (8.2%), and α-terpineol (5.8%) showed good larvicidal activity with 24-h LC_50_ = 17.3 μg/mL against both *Ae. aegypti* and *Cx. quinquefasciatus*, and good antibacterial activity against *E. faecalis* (minimum inhibitory concentration (MIC) = 16 μg/mL) The 48-h larvicidal activities of *M. leucadendra* leaf essential oil, rich in α-eudesmol (17.6%), guaiol (10.9%), linalool (5.1%), (*E*)-caryophyllene (7.0%), and bulnesol (3.6%) were particularly notable, with LC_50_ of 1.4 and 1.8 μg/mL on *Ae. aegypti* and *Cx. quinquefasciatus*. Similarly, *M. leucadendra* bark essential oil, with α-eudesmol (24.1%) and guaiol (11.3%), showed good antibacterial activity against. *E. faecalis*. Both *B. frutescens* and *C. citrinus* leaf essential oils demonstrated anti-*Candida* activities with MIC values of 16 μg/mL. The results of this investigation suggest that essential oils derived from the Myrtaceae may serve as “green” alternatives for the control of mosquitoes and/or complementary antimicrobial agents.

## 1. Introduction

Mosquitoes are important vectors of diseases and kill more humans than any other animal. *Aedes aegypti* (L.) and *Ae. albopictus* (Skuse) (Diptera: Culicidae) are vectors of the yellow fever, dengue, Zika, and chikungunya viruses [1,2,3]; *Culex quinquefasciatus* (Say) is the primary vector of the Saint Louis encephalitis and West Nile viruses, as well as the filarial nematode *Wuchereria bancrofti*, and may also be a vector of the Zika virus [4].

Microbial infections continue to be a problem, for humans [5], as well as for livestock and other agriculture settings [6,7,8]. Compounding this problem are newly emerging pathogenic microorganisms, in addition to re-emerging multidrug-resistant pathogens [9,10].

The Myrtaceae is comprised of 131 genera and around 5500 species, all of which are woody trees or shrubs and contain essential oils [11]. Several members of the family are commercially important for their medicinal essential oils, such as clove (*Syzygium aromaticum* (L.) Merr. & L.M. Perry), tea tree (*Melaleuca alternifolia* Cheel), allspice (*Pimenta dioica* (L.) Merr.), and *Eucalyptus*. In this work, we present the essential oil compositions of four species of Myrtaceae growing wild in central Vietnam, their larvicidal activities against *Ae. aegypti*, *Ae. albopictus*, and *Cx. quinquefasciatus*, and their antimicrobial activities against *Enterococcus faecalis*, *Staphylococcus aureus*, and *Candida albicans*.

*Baeckea frutescens* L. (syn. *Baeckea chinensis* Gaertn., *Baeckea cochinchinensis* Blume, *Baeckea sumatrana* Blume) is a shrub or small tree that ranges throughout southeastern China (including the provinces of Fujian, Guangdong, Guangxi, Hainan, Jaingxi, and Zhejiang), Burma, Cambodia, India, the Philippines, Thailand, and Vietnam [12].

*Callistemon citrinus* (Curtis) Skeels (syn. *Melaleuca citrina* (Curtis) Dum. Cours., *Callistemon lanceolatus* DC., *Callistemon lanceolatus* Sweet, *Metrosideros citrinus* Curtis, *Metrosideros lanceolata* Sm.) is a shrub or small tree, native to Australia, but has been introduced to tropical and subtropical regions worldwide [13].

*Melaleuca leucadendra* (L.) L. (syn. *Melaleuca viridiflora* C.F. Gaertn., *Myrtus leucadendra* L.) is a tree growing as large as 40 m in height, native to tropical Australia (Queensland, Northern Territory, and Western Australia, New Guinea, and islands of eastern Indonesia [14]. The tree has been introduced to other tropical areas [12], including Vietnam, where it is grown for use as poles and construction materials [14].

*Syzygium nervosum* DC. (syn. *Cleistocalyx operculatus* (Roxb.) Merr. & L.M.Perry, *Eugenia operculata* Roxb.) is a medium-sized tree native to the Asian tropics, from southern China (Guangdong, Guangxi, Hainan, Xizang Zizhiqu, and Yunnan provinces), India, Burma, Sri Lanka, Thailand, and Vietnam [12], and south into eastern Australia [15].

**Figure 1:**
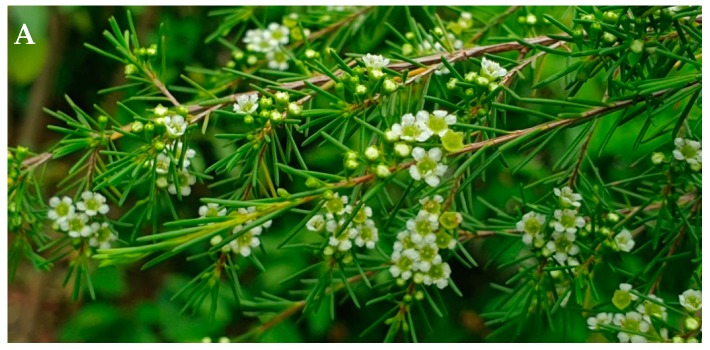

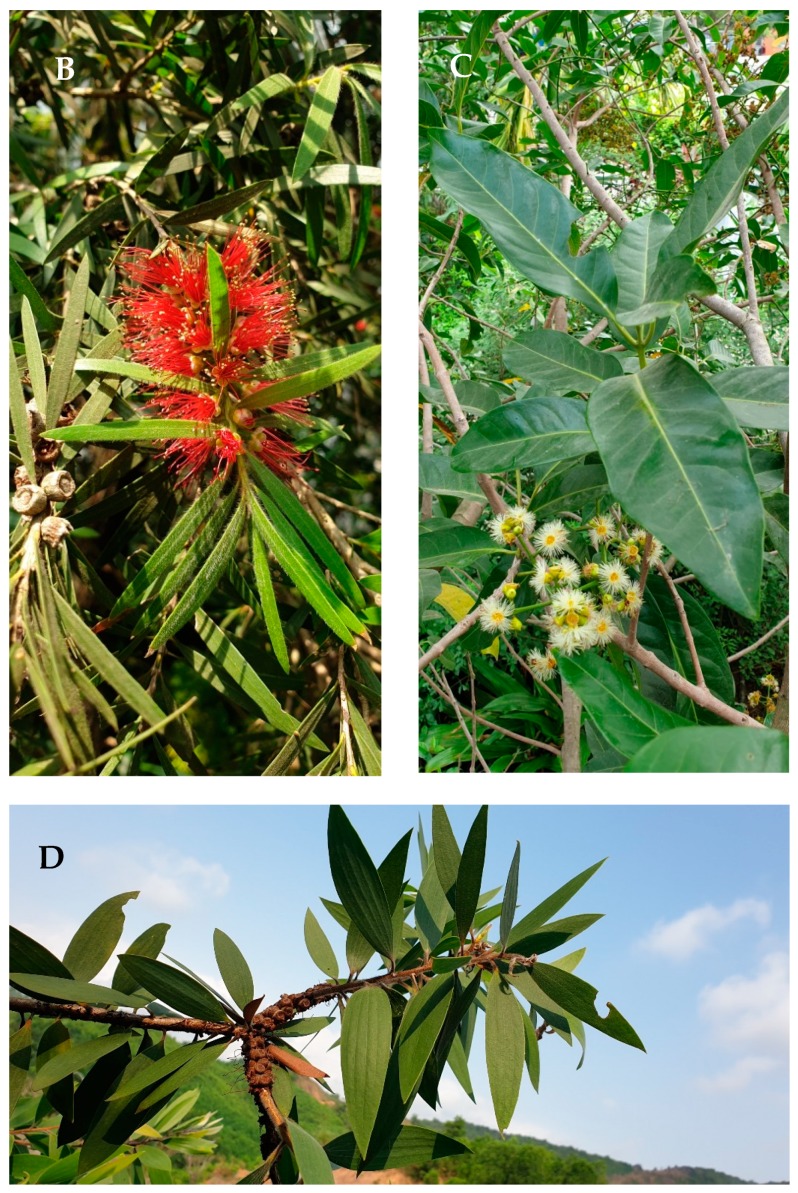
Photographs of the plants examined in this work. **A:** *Baeckea frutescens*, **B:** *Callistemon citrinus*, **C:** *Syzygium nervosum*, **D:** *Melaleuca leucadendra*.

Photographs of the plants presented in this work are shown in Figure 1.

## 2. Results and Discussion

### 2.1. Chemical Compositions

The essential oil from the fresh leaves of *Baeckea frutescens* was obtained in a yield of 2.23%. The leaf essential oil composition of *B. frutescens* is presented in Table 1. A total of 88 compounds were identified accounting for 100% of the essential oil composition, with monoterpene hydrocarbons (55.6%) predominating. The major components were α-pinene (11.1%), β-pinene (19.0%), *p*-cymene (8.9%), 1,8-cineole (10.1%), γ-terpinene (11.7%), (*E*)-caryophyllene (7.1%), and α-humulene (9.9%). Leaf essential oil compositions have previously been reported from Vietnam [16,17,18], China [19], and from Malaysia [20]. The compositions of these essential oils have shown remarkable chemical variation. Nevertheless, the composition of *B. frutescens* in this present study is very similar to that found in a sample collected from Đồng Hới, Quảng Bình Province [16], and sample 2 (from Sóc Sơn District, Hanoi) reported by Tam and co-workers [17]. 

**Figure 2:**
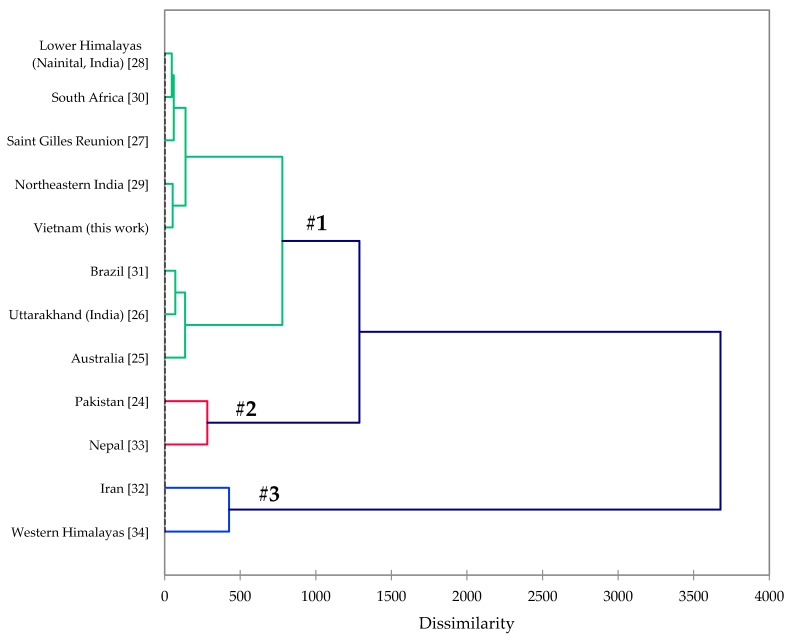
Dendrogram obtained from the agglomerative hierarchical cluster analysis of *Callistemon citrinus* leaf essential oil compositions.

The leaf and fruit essential oils of *Callistemon citrinus* were obtained in yields of 0.62% and 0.34%, respectively. A total of 53 compounds were identified in the leaf essential oil of *C. citrinus*, and 63 compounds were identified in the fruit essential oil, accounting for 99.6% and 99.4% of the compositions, respectively. Monoterpene hydrocarbons (27.6% and 53.8%) and oxygenated monoterpenoids (69.9% and 41.3%) dominated the leaf and fruit oils, respectively. The major components in *C. citrinus* leaf and fruit essential oils were α-pinene (18.1% and 35.1%, respectively), limonene (5.4% and 8.2%), 1,8-cineole (56.3% and 32.4%), and α-terpineol (11.2% and 5.8%) (Table 2). There have been several previous examinations of the composition of *C. citrinus* leaf essential oil from various geographical locations [24,25,26,27,28,29,30,31,32,33,34]. An agglomerative hierarchical cluster analysis based on the compositions of the leaf essential oils (Figure 2) reveals three well-defined clusters: (#1) 1,8-cineole >> α-pinene > α-terpineol, (#2) 1,8-cineole > α-terpineol >> eugenol, and (#3) α-pinene > 1,8-cineole > α-terpineol. The *C. citrinus* leaf essential oil from Vietnam (this study) falls into cluster #1.

Essential oils were obtained from six different tissues of *Melaleuca leucadendra*, young leaves, old leaves, stem bark, fruits, and branch tips, in yields of around 1%. A total of 104 compounds were identified in the *M. leucadendra* essential oils. Sesquiterpene hydrocarbons (18.8%–31.0%) and oxygenated sesquiterpenoids (35.6%–69.5%) were the dominant chemical classes. The essential oil compositions are compiled in Table 3.

Brophy has described two different chemotypes of *M. leucadendra* from Australia, based on leaf essential oil composition [14]. Chemotype I, from Western Australia, is rich in monoterpenoids, e.g., 1,8-cineole (10–45%), *p*-cymene (5–22%), α-pinene (4–19%), limonene (3–6%), and α-terpineol (6–9%). Chemotype II, from eastern Australia, is dominated by phenylpropanoids, which was divided into two subtypes: IIa, eugenol methyl ether (95%–97%), and IIb, (*E*)-*iso*-eugenol methyl ether (74%–88%) subtype). Chemotype IIa has also been represented by samples from Minas Gerais, Brazil [35], and from Lahore, Pakistan [36]. There is a third chemotype, dominated by (*E*)-nerolidol (>90%), which has been described from Uttarakhand, India [37] and from Pernambuco, Brazil [38]. Chemotype I has also been found in Cuba [39] and Rio de Janeiro, Brazil [40]. They were both dominated by 1,8-cineole (43.0% and 48.7%, respectively), but these two samples were also rich in viridiflorol (24.2% and 27.8%, respectively), and therefore, may represent a subtype of chemotype I. 

**Figure 3:**
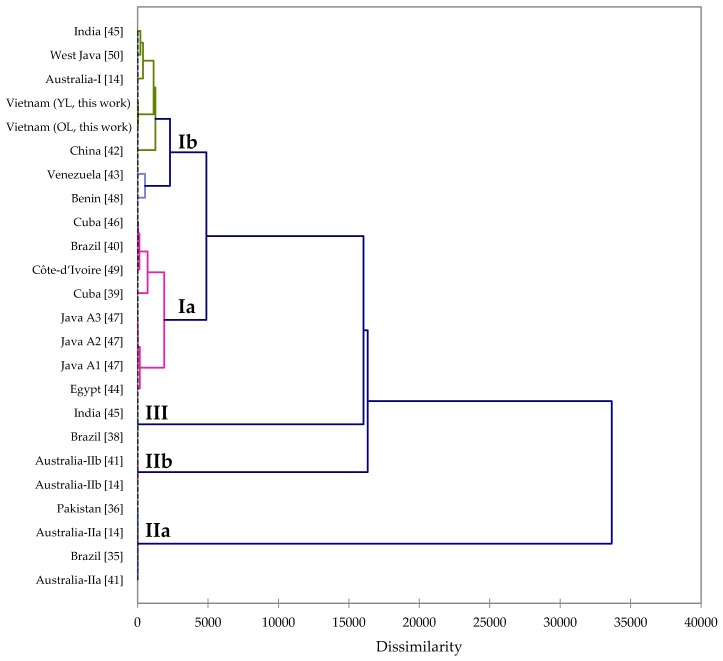
Dendrogram obtained from the agglomerative hierarchical cluster analysis of *Melaleuca leucadendra* leaf essential oil compositions.

An agglomerative hierarchical cluster analysis was carried out using the *M. leucadendra* leaf essential oil compositions reported in the literature [14,36,37,38,39,40,41,42,43,44,45,46,47,48,49,50] (Figure 3). The cluster analysis reveals two sub-types of chemotype I, the two sub-types of chemotype II, as described by Brophy [14], and chemotype III, the nerolidol chemotype. The leaf essential oils of *M. leucadendra* from Vietnam, fall into sub-type Ib; the leaf oils were rich in α-eudesmol (17.6%–21.2%), guaiol (10.9%–12.5%), with lesser concentrations of linalool (4.9%–5.1%), (*E*)-caryophyllene (3.8%–7.0%), and bulnesol (3.6%–5.3%). Concentrations of 1,8-cineole were low (0.0%–5.2%), and (*E*)-nerolidol and viridiflorol were not observed at all.

The leaf essential oil of *Syzygium nervosum* was obtained in 0.2% yield. A total of 61 compounds were identified in the leaf oil of *S. nervosum*, accounting for 90.9% of the composition, with 31.7% monoterpene hydrocarbons, 24.3% sesquiterpene hydrocarbons, and 27.9% oxygenated sesquiterpenoids predominating. The leaf essential oil of *S. nervosum* was rich in (*Z*)-β-ocimene (20.3%), caryophyllene oxide (13.2%), (*E*)-caryophyllene (12.1%), and α-pinene (5.2%) (Table 4). The leaf essential oil composition is qualitatively similar, but quantitatively different, to a previous report on the leaf essential oil from Lê Mao District, Vinh City, Vietnam [51]. Both samples had relatively high concentrations of α-pinene, (*Z*)-β-ocimene, (*E*)-β-ocimene, and (*E*)-caryophyllene (3.7%, 32.1%, 9.4%, and 14.5%, respectively, in the Vinh City sample), but the concentration of myrcene was much higher (24.6%) in the sample from Vinh City. The leaf essential oil *S. nervosum* from Nepal showed a very different composition with myrcene (69.7%), (*E*)-β-ocimene (12.2%), (*Z*)-β-ocimene (4.8%), and linalool (4.1%) [52].

### 2.2. Mosquito Larvicidal Activity

The 24-h and 48-h larvicidal activities are presented in Table 5 and Table 6, respectively. The Myrtaceae essential oils presenting the best 24-h larvicidal activities were *C. citrinus* fruit essential oil (LC_50_ = 17.3 μg/mL against both *Ae. aegypti* and *Cx. quinquefasciatus*), *M. leucadendra* stem bark essential oil (LC_50_ = 17.1, 19.3, and 21.4 μg/mL against *Ae. aegypti*, *Ae. albopictus*, and *Cx. quinquefasciatus*, respectively), *M. leucadendra* fruit essential oil (LC_50_ = 13.9, 19.2, and 26.2 μg/mL against *Ae. aegypti*, *Ae. albopictus*, and *Cx. quinquefasciatus*, respectively), and, especially, *M. leucadendra* old leaf essential oil (LC_50_ = 7.4 and 6.6 μg/mL against *Ae. aegypti* and *Cx. quinquefasciatus*, respectively). The 48-h larvicidal activities of *M. leucadendra* old leaf essential oil are particularly notable with LC_50_ of 1.4 and 1.8 μg/mL on *Ae. aegypti* and *Cx. quinquefasciatus*.

The larvicidal activities of *M. leucadendra* essential oils are likely due to the high concentrations of α-eudesmol and guaiol, or possibly synergistic effects involving these compounds. Unfortunately, there appear to be no reports on the larvicidal activities of these compounds.

It is tempting to suggest that the sensitivity of mosquito larvae to *C. citrinus* fruit essential oil is due to the combination of α-pinene and 1,8-cineole. 1,8-Cineole, (+)-α-pinene, and (–)-α-pinene have been screened against *Ae. aegypti* larvae, and showed modest larvicidal activities (LC_50_) of 74.9, 50.9, and 64.8 μg/mL, respectively [53]. Furthermore, *Hedychium bousigonianum* cv. “Tai Emperor” rhizome essential oil, with 16.7% α-pinene and 25.5% 1,8-cineole, showed only marginal larvicidal activity against *Ae. aegypti* (80% lethality at 125 μg/mL) [54]. In addition, Pavela has shown that α-pinene has marginal larvicidal activity against *Cx. quinquefasciatus* (LC_50_ = 95 μg/mL), 1,8-cineole is inactive (LC_50_ > 250 μg/mL), and a binary mixture of the two compounds does not demonstrate synergistic activity [55]. The observed larvicidal activities of *C. citrinus* fruit essential oil is apparently due to synergistic activities involving minor components. It has been shown that *Musca domestica* preferentially metabolizes the major components in an essential oil, which leaves the components of lower concentrations to act as the toxic agents [56].

*Baeckea frutescens* and *Callistemon citrinus* leaf essential oils were relatively inactive against *Cx. quinquefasciatus*, with 24-h LC_50_ values of 81.7 μg/mL and 73.6 μg/mL, respectively. However, both of those essential oils showed high concentrations of α-pinene (11.1% and 18.1%, respectively) and 1,8-cineole (10.1% and 56.3%, respectively). The leaf oil of *B. frutescens* also had high concentrations of β-pinene (19.0%), γ-terpinene (11.7%), α-humulene (9.9%), and (*E*)-caryophyllene (7.1%). The relative inactivity of *B. frutescens* against *Cx. quinquefasciatus* is difficult to explain. Both β-pinene and γ-terpinene have shown good larvicidal activity against *Cx. pipiens pallens* with 24-h LC_50_ of 21.1, 12.9, and 12.6 μg/mL for (+)-β-pinene, (–)-β-pinene, and γ-terpinene, respectively [53]. (*E*)-Caryophyllene showed only weak larvicidal activity (LC_50_ = 93.7 μg/mL), however [53], and α-humulene was found to be inactive against this mosquito [57]. The major components of *C. citrinus* leaf essential oil and *C. citrinus* fruit essential oil are qualitatively similar. It is not obvious why the larvicidal activities of these two oils against *Cx. quinquefasciatus* are so different, but it may be due to synergistic effects of minor components present in the fruit essential oil but absent in the leaf essential oil. Apparently, there is more involved in the larvicidal activities of these essential oils than the major components.

*Syzygium nervosum* essential oil larvicidal activity is also difficult to explain. There were high concentrations of (*Z*)-β-ocimene (20.3%), (*E*)-caryophyllene (12.1%), and caryophyllene oxide (13.2%). Unfortunately, we have found no larvicidal screening of (*Z*)-β-ocimene in the literature. Note, however, that *Syzygium jambolana* essential oil, rich in (*Z*)-β-ocimene (27.2%), was inactive against *Ae. aegypti* larvae (LC_50_ = 433 μg/mL) [58]. Furthermore, (*E*)-caryophyllene and caryophyllene oxide have shown only marginal larvicidal activities against *Ae. aegypti* or *Cx. pipiens pallens* [53,57].

### 2.3. Antimicrobial Activity

The Myrtaceae essential oils were screened for antibacterial activity against *Enterococcus faecalis* (ATCC 29912) and *Staphylococcus aureus* (ATCC 25923), and for antifungal activity against *Candida albicans* (ATCC 10231). The antimicrobial activities are summarized in Table 7.

The leaf essential oils of *B. frutescens* and *C. citrinus* both showed excellent anti-*Candida* activity, with minimum inhibitory concentration (MIC) values of 16 μg/mL. van Zyl and co-workers have screened several monoterpenoids against *C. albicans*, and many of the major components that were found in *B. frutescens* and *C. citrinus* leaf essential oils did show notable activities, including α-pinene (MIC 12.0 μg/mL), β-pinene (MIC 1.0 μg/mL), limonene (MIC 10.0 μg/mL), and γ-terpinene (MIC 6.0 μg/mL) [59]. 1,8-Cineole and α-terpineol are relatively inactive against *C. albicans*, however [60,61]. A perusal of the literature reveals a broad spectrum of reported antimicrobial activities for terpenoid constituents against *E. faecalis*, *S. aureus*, and *C. albicans* (Table 8). There are several potential reasons for the apparent discrepancies, including variation in antimicrobial assay protocols, different susceptibilities of different strains of a particular microorganism, mathematical errors in calculating dilutions and MIC values.

*Callistemon citrinus* fruit essential oil, dominated by α-pinene (35.1%) and 1,8-cineole (32.4%), was particularly active against *E. faecalis*. Neither of these compounds have shown notable activity against *E. faecalis*, however (Table 8); the activity observed for *C. citrinus* fruit essential oil must be attributed to synergistic activity of less abundant components. *Melaleuca leucadendra* bark essential oil, which was rich in α-eudesmol (24.1%) and guaiol (11.3%), also exhibited notable activity against *E. faecalis*, possibly due to the high concentrations of sesquiterpene alcohols present.

## 3. Materials and Methods 

### 3.1. Plant Collection

Plant materials were collected from wild-growing plants in the Hoa Vang and Hoa Khanh districts of Da Nang city. The plants were identified by Do Ngoc Dai. In each case, the fresh plant material was chopped, and 2.0 kg was subjected to hydrodistillation using a Clevenger-type apparatus (Table 9).

### 3.2. Gas Chromatographic – Mass Spectral Analysis

Each of the essential oils was analyzed by gas chromatography-mass spectrometry (GC-MS), as previously reported [81], using a Shimadzu GCMS-QP2010 Ultra, fitted with a ZB-5 column. Identification of the oil components was based on their retention indices determined by reference to a homologous series of *n*-alkanes, and by comparison of their mass spectral fragmentation patterns with those in the NIST [21] and FFSNC [22] databases and our own Sat-Set library [23].

### 3.3. Mosquito Larvicidal Assays

Mosquito colonies of *Aedes aegypti*, *Aedes albopictus*, and *Culex quinquefasciatus* were obtained and maintained as previously described [82].

Larvicidal activities of the essential oils were evaluated according to the protocol of Liu and co-workers [83] with slight modifications. For each assay, 150 mL of water containing 20 fourth-instar mosquito larvae was placed into 250-mL beakers and aliquots of the essential oils dissolved in EtOH (1% stock solution) were then added. A set of controls using EtOH only (negative control) and permethrin (positive control) were included for comparison. Mortality was recorded after 24 h and after 48 h of exposure, during which no nutritional supplement was added. The experiments were carried out at 25 ± 2 °C. Each test was conducted in quadruplicate with five concentrations (100, 50, 25, 12.5 and 6 μg/mL). The data obtained were subjected to log-probit analysis [84] to obtain LC_50_ values, LC_90_ values and 95% confidence limits using Minitab^®^ 19 (Minitab, LLC, State College, PA, USA).

### 3.4. Antimicrobial Screening

The antimicrobial activity of the essential oils was evaluated using two bacteria (*Enterococcus faecalis*, ATCC 299212, and *Staphylococcus aureus*, ATCC 25923) and one yeast (*Candida albicans*, ATCC 10231) using the microdilution broth susceptibility assay, as previously reported [82]. Stock solutions of the each of the essential oils were prepared in dimethylsulfoxide. Dilution series were prepared from 16,384 to 2 μg/mL (2^14^, 2^13^, 2^12^, 2^11^, 2^10^, 2^9^, 2^7^, 2^5^, 2^3^ and 2^1^ µg/mL) in sterile distilled water in micro-test tubes from where they were transferred to the 96-well microtiter plates for the assays.

### 3.5. Agglomerative Hierarchical Cluster Analysis

The essential oil compositions from this work and from the published literature were treated as operational taxonomic units (OTUs). The percentage composition of the major components of the essential oils was used to determine the chemical relationship between the various essential oil samples by agglomerative hierarchical cluster (AHC) analysis, using the XLSTAT software, version 2018.1.1.6097 (Addinsoft™, Paris, France). Euclidean distance was used to measure dissimilarity, and Ward’s method was used for cluster definition.

## 4. Conclusions

Essential oils derived from *Baeckea frutescens*, *Callistemon citrinus*, *Melaleuca leucadendra*, and *Syzygium nervosum* have shown larvicidal activities against the mosquito species tested. In most cases, the larvicidal activities cannot be attributed to the major components, and synergistic interactions with minor components are likely responsible. Likewise, all of the Myrtaceae essential oils examined for antimicrobial activity showed promise. Thus, these essential oils may serve as “green” vector control agents and/or complementary antimicrobial agents, as well as providing value-added commodities for harvested timbers (e.g., *Melaleuca leucadendra*).

## Figures and Tables

**Table 1:** Chemical composition of *Baeckea frutescens* leaf essential oil from central Vietnam.

RI_(calc)_	RI_(db)_	Compounds	%	RI_(calc)_	RI_(db)_	Compounds	%
922	927	α-Thujene	1.8	1370	1375	α-Copaene	0.2
930	933	α-Pinene	11.1	1398	1405	(*Z*)-Caryophyllene	tr
943	948	α-Fenchene	tr	1401	1406	α-Gurjunene	tr
945	953	Camphene	0.1	1415	1417	(*E*)-Caryophyllene	7.1
968	972	Sabinene	tr	1433	1438	Aromadendrene	0.1
975	978	β-Pinene	19.0	1452	1454	α-Humulene	9.9
984	991	Myrcene	0.3	1455	1457	*allo*-Aromadendrene	0.1
1000	1004	*p*-Mentha-1(7),8-diene	tr	1466	1472	*trans*-Cadina-1(6),4-diene	0.1
1003	1007	α-Phellandrene	0.1	1469	1478	γ-Muurolene	tr
1005	1009	δ-3-Carene	tr	1483	1487	β-Selinene	0.1
1013	1018	α-Terpinene	0.3	1485	1490	γ-Amorphene	tr
1021	1025	*p*-Cymene	8.9	1490	1501	α-Selinene	0.1
1025	1030	Limonene	1.7	1492	1497	α-Muurolene	0.1
1029	1030	1,8-Cineole	10.1	1500	1507	Geranyl isobutyrate	0.1
1030	1034	(*Z*)-β-Ocimene	tr	1506	1512	γ-Cadinene	0.2
1041	1045	(*E*)-β-Ocimene	tr	1509	1519	Cubebol	tr
1055	1057	γ-Terpinene	11.7	1512	1518	δ-Cadinene	0.9
1065	1069	*cis*-Linalool oxide (furanoid)	tr	1515	1519	*trans*-Calamenene	0.1
1081	1086	Terpinolene	0.7	1516	1521	Zonarene	0.1
1085	1093	*p*-Cymenene	tr	1526	1536	*trans*-Cadine-1,4-diene	0.1
1096	1101	Linalool	4.4	1530	1538	α-Cadinene	tr
1098	1104	Hotrienol	tr	1534	1544	α-Calacorene	tr
1114	1119	*endo*-Fenchol	0.1	1541	1549	α-Elemol	tr
1133	1139	Nopinone	tr	1545	1551	(*Z*)-Caryphyllene oxide	0.1
1136	1141	*trans*-Pinocarveol	tr	1554	1562	(*E*)-Nerolidol	0.5
1150	1156	Camphene hydrate	tr	1570	1576	Spathulenol	tr
1165	1170	δ-Terpineol	0.1	1576	1587	Caryophyllene oxide	2.0
1167	1170	Borneol	0.1	1579	1590	Globulol	0.1
1169	1171	*cis*-Linalool oxide (pyranoid)	tr	1592	1592	Humulene epoxide I	0.3
1173	1179	2-Isopropenyl-5-methyl-4-hexenal	0.1	1598	1605	Ledol	0.1
1176	1180	Terpinen-4-ol	0.7	1604	1613	Humulene epoxide II	2.4
1178	1188	Naphthalene	tr	1619	1624	Muurola-4,10(14)-dien-1β-ol	tr
1181	1186	*p*-Cymen-8-ol	tr	1621	1628	1-*epi*-Cubenol	0.3
1190	1195	α-Terpineol	1.7	1625	1611	Germacra-1(10),5-dien-4α-ol	0.3
1198	1203	*p*-Cumenol	tr	1626	1632	Humulenol II	0.3
1219	1229	Nerol	tr	1630	1636	Caryophylla-4(12),8(13)-dien-5β-ol	0.1
1234	1240	Ascaridole	tr	1634	1643	τ-Cadinol	0.2
1244	1244	Geraniol	0.1	1636	1645	τ-Muurolol	0.1
1261	1268	Geranial	tr	1639	1651	α-Muurolol (= δ-Cadinol)	0.1
1268	1275	*trans*-Ascaridol glycol	tr	1648	1652	α-Eudesmol	0.5
1274	1284	*p*-Cymen-7-ol	tr	1841	1837	Homoisobaeckeol	0.5
1284	1289	Thymol	tr			Monoterpene hydrocarbons	55.6
1291	1399	Carvacrol	tr			Oxygenated monoterpenoids	17.5
1298	1306	Isoascaridole	tr			Sesquiterpene hydrocarbons	19.1
1314	1320	Methyl geranate	0.1			Oxygenated sesquiterpenoids	7.3
1341	1349	α-Cubebene	tr			Benzenoid aromatics	0.5
1344	1357	Eugenol	tr			Others	tr
						Total identified	100.0

RI_(calc)_: Retention indices determined with respect to a homologous series of *n*-alkanes on a ZB-5ms column. RI_(db)_: Retention indices obtained from the databases [21,22,23]. tr: trace (< 0.05%).

**Table 2:** Chemical compositions of the leaf and fruit essential oils of *Callistemon citrinus* from central Vietnam.

RI_(calc)_	RI_(db)_	Compound	% Composition	
			Leaf	Fruit
793	791	2,4-Dimethyl-3-pentanone	0.3	tr
912	913	Isobutyl isobutyrate	0.2	0.3
924	927	α-Thujene	0.3	0.8
932	933	α-Pinene	18.1	35.1
946	948	α-Fenchene	tr	tr
948	953	Camphene	0.1	0.1
971	972	Sabinene	tr	tr
976	978	β-Pinene	0.6	0.7
987	989	Myrcene	0.1	0.5
999	1000	δ-2-Carene	tr	0.1
1004	1004	p-Mentha-1(7),8-diene	0.1	0.1
1006	1007	α-Phellandrene	0.4	1.6
1008	1009	δ-3-Carene	0.1	0.1
1011	1014	Isoamyl isobutyrate	0.2	0.3
1014	1018	α-Terpinene	---	0.2
1014	1015	2-Methylbutyl isobutyrate	tr	0.1
1021	1022	Ethyl 3-methylbut-3-enyl carbonate	0.1	0.1
1024	1025	*p*-Cymene	2.2	4.6
1029	1030	Limonene	5.4	8.2
1030	1032	1,8-cineole	56.3	32.4
1032	1034	(*Z*)-β-Ocimene	---	0.1
1044	1046	(*E*)-β-Ocimene	tr	0.2
1051	1050	Prenyl isobutyrate	tr	0.1
1057	1057	γ-Terpinene	0.3	1.0
1084	1087	Terpinolene	0.1	0.6
1088	1093	*p*-Cymenene	---	0.1
1099	1101	Linalool	0.5	1.4
1119	1119	*endo*-Fenchol	0.1	0.1
1140	1141	*trans*-Pinocarveol	0.3	tr
1155	1156	Camphene hydrate	tr	tr
1163	1164	Pinocarvone	tr	---
1170	1170	δ-Terpineol	0.2	0.1
1170	1165	*iso*-Borneol	---	0.1
1173	1173	Borneol	0.1	0.1
1179	1179	2-Isopropenyl-5-methyl-4-hexenal	0.1	tr
1180	1180	Terpinen-4-ol	0.5	0.6
1185	1188	Naphthalene	0.1	---
1186	1189	*p*-Cymen-8-ol	---	tr
1188	1187	*trans-p*-Mentha-1(7),8-dien-2-ol	0.1	---
1194	1195	α-Terpineol	11.2	5.8
1202	1202	*cis*-Sabinol	---	0.1
1219	1223	*trans*-Carveol	0.1	tr
1230	1230	*cis-p*-Mentha-1(7),8-dien-2-ol	tr	---
1249	1249	Geraniol	0.5	0.6
1298	1300	Carvacrol	tr	0.1
1351	1356	Eugenol	0.1	0.1
1385	1390	β-Elemene	---	0.1
1392	1395	Phenylethyl isobutyrate	tr	tr
1417	1417	(*E*)-Caryophyllene	0.1	0.2
1436	1438	Aromadendrene	0.1	0.2
1452	1454	α-Humulene	---	0.1
1458	1458	*allo*-Aromadendrene	0.1	0.1
1477	1480	Germacrene D	---	tr
1487	1491	Viridiflorene	---	0.1
1500	1503	(*E*,*E*)-α-Farnesene	---	0.1
1505	1507	Geranyl isobutyrate	0.1	---
1505	1508	β-Bisabolene	---	0.1
1514	1518	δ-Cadinene	---	tr
1535	1539	Flavesone	0.3	0.3
1557	1561	(*E*)-Nerolidol	---	0.1
1575	1578	Spathulenol	0.4	1.3
1580	1577	Caryophyllene oxide	tr	0.1
1584	1590	Globulol	0.1	0.2
1593	1594	Viridiflorol	0.1	0.1
1595	1599	Cubeban-11-ol	tr	0.1
1609	1614	*iso*-Leptospermone	tr	0.1
1619	1626	Leptospermone	tr	0.2
1629	1629	*iso*-Spathulenol	---	0.2
		Monoterpene hydrocarbons	27.6	53.8
		Oxygenated monoterpenoids	69.9	41.3
		Sesquiterpene hydrocarbons	0.2	0.8
		Oxygenated sesquiterpenoids	0.5	2.0
		Others	1.4	1.4
		Total identified	99.6	99.4

RI_(calc)_: Retention indices determined with respect to a homologous series of *n*-alkanes on a ZB-5ms column. RI_(db)_: Retention indices obtained from the databases [21,22,23]. tr: trace (<0.05%).

**Table 3:** Chemical compositions of essential oils from *Melaleuca leucadendra* from central Vietnam.

RI_(calc)_	RI_(db)_	Compound	% Composition				
			Young Leaf	Old Leaf	Stem Bark	Fruit	Branch Tips
923	927	α-Thujene	0.8	0.4	0.1	tr	1.2
931	933	α-Pinene	0.7	0.6	0.8	0.2	1.4
947	953	Camphene	---	tr	tr	tr	---
960	960	Benzaldehyde	0.1	0.1	---	---	tr
975	978	β-Pinene	0.1	0.2	0.3	0.1	0.1
987	991	Myrcene	0.2	0.3	0.2	0.1	0.2
1003	1004	*p*-Mentha-1(7),8-diene	---	---	tr	---	---
1005	1007	α-Phellandrene	0.3	0.2	---	---	0.3
1007	1009	δ-3-carene	0.1	tr	tr	---	0.1
1015	1018	α-Terpinene	0.4	0.3	---	---	0.4
1023	1025	*p*-Cymene	3.9	1.7	1.3	0.5	8.7
1027	1030	Limonene	0.3	0.8	1.4	0.4	0.7
1029	1031	β-Phellandrene	tr	0.1	tr	---	0.1
1030	1030	1,8-cineole	---	5.2	1.8	0.2	tr
1033	1034	(*Z*)-β-Ocimene	---	tr	---	---	tr
1043	1045	(*E*)-β-Ocimene	---	tr	---	---	tr
1056	1057	γ-Terpinene	2.2	1.3	tr	---	3.3
1068	1069	*cis*-Linalool oxide (furanoid)	---	---	---	---	tr
1084	1086	Terpinolene	3.0	1.6	0.1	tr	4.4
1089	1093	*p*-Cymenene	0.1	tr	tr	---	0.2
1099	1101	Linalool	4.9	5.1	1.4	0.4	4.2
1103	1107	Nonanal	---	---	0.1	---	---
1110	1110	1,3,8-*p*-Menthatriene	tr	tr	---	---	---
1122	1124	*cis-p*-Menth-2-en-1-ol	tr	tr	---	---	---
1141	1142	Epoxyterpinolene	0.3	tr	---	---	0.6
1147	1149	*iso*-Pulegol	---	tr	---	---	tr
1168	1170	δ-Terpineol	---	tr	---	---	---
1170	1170	Borneol	---	tr	---	---	---
1177	1179	2-Isopropenyl-5-methyl-4-hexenal	0.2	0.1	---	---	0.3
1179	1180	Terpinen-4-ol	0.9	0.4	tr	tr	1.1
1183	1188	Naphthalene	---	---	0.1	0.1	0.2
1184	1188	4’-Methylacetophenone	0.1	tr	---	---	0.1
1186	1188	*p*-Cymen-8-ol	1.0	0.2	0.1	0.1	1.2
1194	1195	α-Terpineol	0.7	1.8	0.5	0.1	0.6
1198	1195	*p*-Menth-3-en-7-al	---	---	---	---	0.1
1202	1203	*p*-Cumenol	0.1	0.1	---	---	0.1
1222	1222	*iso*-Ascaridol	---	tr	---	---	0.1
1223	1226	Nerol	---	tr	tr	---	---
1225	1227	Citronellol	---	tr	tr	tr	0.1
1248	1249	Geraniol	0.2	0.6	0.4	0.1	0.2
1266	1266	Geranial	---	tr	tr	---	---
1273	1275	*trans*-Ascaridol glycol	0.2	tr	---	---	0.1
1290	1291	*cis*-Ascaridol glycol	0.1	---	---	---	0.1
1293	1305	Benzophenone	----	tr	---	---	---
1318	1318	3-Hydroxycineole	0.2	---	---	---	0.1
1348	1356	Eugenol	---	0.1	---	---	---
1367	1371	α-Ylangene	0.4	0.6	0.9	0.6	0.7
1373	1375	α-Copaene	0.2	0.3	0.8	0.3	0.3
1375	1380	Geranyl acetate	---	0.1	0.2	tr	0.1
1381	1382	β-Bourbonene	---	---	tr	---	---
1387	1390	β-Elemene	0.1	0.1	0.1	tr	0.1
1389	1394	Sativene	0.1	0.1	0.1	tr	0.1
1401	1405	(*Z*)-Caryophyllene	---	---	tr	tr	---
1417	1417	(*E*)-Caryophyllene	3.8	7.0	5.5	4.3	5.7
1421	1428	8-Hydroxycarvotanacetone	0.1	---	---	---	0.1
1426	1427	γ-Elemene	0.2	0.3	0.1	0.1	0.1
1432	1436	α-Guaiene	0.1	0.2	0.2	0.2	0.2
1438	1444	Guaia-6,9-diene	0.2	0.2	0.1	0.1	0.2
1444	1448	*cis*-Muurola-3,5-diene	0.2	0.2	0.1	0.1	0.2
1446	1447	*iso*-Germacrene D	0.1	0.2	0.1	0.1	0.2
1453	1454	α-Humulene	2.8	4.4	3.5	2.8	3.7
1467	1473	Drima-7,9(11)-diene	0.1	0.2	0.2	0.2	0.2
1470	1476	Selina-4,11-diene	0.2	0.5	0.5	0.4	0.6
1474	1476	γ-Gurjunene	0.6	1.1	1.1	0.9	1.4
1476	1479	α-Amorphene	0.7	1.2	1.5	0.9	1.2
1484	1488	δ-Selinene	1.0	1.6	0.7	0.6	1.3
1487	1492	β-Selinene	2.4	3.7	4.8	3.1	4.2
1490	1490	γ-Amorphene	0.2	0.3	0.4	0.3	0.5
1494	1501	α-Selinene	2.1	3.7	3.6	2.5	4.1
1495	1496	*trans*-Muurola-4(14),5-diene	---	0.2	---	---	0.1
1496	1497	α-Muurolene	---	---	0.2	0.1	---
1499	1505	α-Bulnesene	---	0.1	0.1	0.2	0.1
1499	1506	δ-Amorphene	---	0.2	---	---	---
1500	1502	*trans*-β-Guaiene	---	0.3	---	---	---
1501	1501	β-Dihydroagarofuran	---	---	0.2	0.2	---
1515	1518	δ-Cadinene	---	---	0.2	0.1	---
1516	1520	7-epi-α-Selinene	---	---	---	0.2	---
1517	1519	*trans*-Calamenene	---	---	0.7	0.4	---
1534	1540	Selina-4(15),7(11)-diene	0.5	0.6	0.6	0.6	0.7
1539	1541	α-Calacorene	0.3	0.6	0.8	0.5	0.6
1539	1546	Selina-3,7(11)-diene	0.3	0.2	---	0.3	0.3
1545	1546	α-Elemol	0.3	0.1	0.3	0.4	---
1556	1557	Germacrene B	0.4	0.4	0.1	---	0.1
1580	1587	Caryophyllene oxide	1.8	2.3	3.3	3.2	4.0
1590	1600	Khusimone	0.2	0.3	0.4	0.3	0.3
1595	1603	Guaiol	12.5	10.9	11.3	10.4	7.3
1607	1613	Humulene epoxide II	0.8	0.9	1.5	1.3	1.6
1610	1609	Rosifoliol	0.5	0.4	0.5	0.5	0.2
1620	1611	Germacra-1(10),5-dien-4α-ol	0.2	0.2	0.2	---	0.2
1623	1624	Selina-6-en-4β-ol	2.0	1.6	1.7	2.2	1.2
1624	1629	*iso*-Spathulenol	0.2	---	---	---	---
1628	1631	Eremoligenol	3.4	3.4	4.9	6.5	2.7
1630	1633	γ-Eudesmol	3.9	2.8	3.5	5.3	1.9
1632	1634	*cis*-Cadin-4-en-7-ol	3.5	3.0	3.3	3.5	2.2
1635	1636	Caryophylla-4(12),8(13)-dien-5β-ol	---	0.2	0.2	0.1	0.2
1638	1645	Hinesol	1.0	0.9	1.2	1.6	0.7
1645	1644	Selina-3,11-dien-6α-ol	---	0.2	0.3	---	0.2
1653	1652	α-Eudesmol	21.2	17.6	24.1	30.7	13.7
1657	1660	Selin-11-en-4α-ol	1.9	1.5	1.3	1.6	1.0
1663	1673	Bulnesol	5.3	3.6	3.3	4.4	2.2
1668	1671	14-Hydroxy-9-*epi*-(*E*)-caryophyllene	---	---	0.5	---	---
1670	1677	Cadalene	---	---	0.3	0.2	---
1695	1696	Juniper camphor	---	0.2	0.1	0.2	0.1
1918	1929	Carissone	---	---	0.1	0.4	---
		Monoterpene hydrocarbons	11.9	7.2	4.2	1.3	21.2
		Oxygenated monoterpenoids	8.8	13.5	4.4	0.8	9.0
		Sesquiterpene hydrocarbons	18.8	30.8	30.5	23.4	31.0
		Oxygenated sesquiterpenoids	56.9	47.6	59.1	69.5	35.6
		Benzenoid aromatics	0.2	0.1	0.0	0.0	0.1
		Others	0.0	0.0	0.2	0.1	0.2
		Total identified	96.6	99.3	98.4	95.2	97.1

RI_(calc)_: Retention indices determined with respect to a homologous series of *n*-alkanes on a ZB-5ms column. RI_(db)_: Retention indices obtained from the databases [21,22,23]. tr: trace (<0.05%).

**Table 4:** Chemical compositions of essential oils from *Syzygium nervosum* from central Vietnam.

RI_(calc)_	RI_(db)_	Compound	%	RI_(calc)_	RI_(db)_	Compound	%
930	933	α-Pinene	5.2	1486	1492	β-Selinene	0.9
968	971	Tetrahydrofurfuryl acetate	0.2	1492	1501	α-Selinene	0.9
975	978	β-Pinene	1.0	1494	1500	α-Muurolene	0.4
986	991	Myrcene	0.4	1509	1512	γ-Cadinene	0.9
1022	1025	*p*-Cymene	0.1	1514	1518	δ-Cadinene	1.0
1027	1030	Limonene	0.2	1533	1538	α-Cadinene	0.4
1033	1034	(*Z*)-β-Ocimene	20.3	1538	1541	α-Calcorene	0.4
1043	1045	(*E*)-β-Ocimene	3.5	1557	1560	(*E*)-Nerolidol	0.1
1089	1091	Rosefuran	0.7	1559	1560	β-Calacorene	0.5
1092	1101	α-Pinene oxide	1.3	1573	1576	Spathulenol	0.6
1097	1101	Linalool	0.3	1579	1587	Caryophyllene oxide	13.2
1101	1102	6-Methyl-3,5-heptadien-2-one	0.5	1582	1590	Globulol	1.2
1125	1127	*allo*-Ocimene	0.8	1591	1592	Viridiflorol	0.4
1127	1128	(*Z*)-Epoxy ocimene (= (*Z*)-Myroxide)	0.5	1593	1593	Guaiol	0.5
1137	1137	(*E*)-Epoxy ocimene (= (*E*)-Myroxide)	0.4	1595	1592	Humulene epoxide I	0.2
1167	1169	Rosefuran epoxide	0.3	1603	1607	β-Oplopenone	0.8
1170	1171	*p*-Mentha-1,5-dien-8-ol	0.2	1606	1613	Humulene epoxide II	1.8
1182	1188	Naphthalene	0.4	1623	1624	Selina-6-en-4β-ol	3.4
1193	1195	α-Terpineol	0.1	1624	1628	1-*epi*-Cubenol	0.6
1199	---	(3*Z*)-Octenyl acetate	0.4	1631	1634	*cis*-Cadin-4-en-7-ol	0.4
1199	1205	*cis*-4-Caranone	0.1	1634	1636	Caryophylla-4(12),8(13)-dien-5β-ol	0.5
1206	1207	(3*E*)-Octenyl acetate	0.7	1638	1643	τ-Cadinol	0.7
1353	1349	α-Terpinyl acetate	0.7	1640	1644	τ-Muurolol	0.2
1366	1367	Cyclosativene	0.2	1643	1651	α-Muurolol (= δ-Cadinol)	0.2
1372	1375	α-Copaene	0.4	1645	1645	Selina-3,11-dien-6α-ol	0.4
1374	1380	Geranyl acetate	0.4	1652	1655	α-Cadinol	1.7
1417	1417	(*E*)-Caryophyllene	12.1	1655	1660	Selin-11-en-4α-ol	0.6
1426	1433	β-Copaene	0.3	1698	1697	(*E*)-*trans*-α-Bergamota-2,10-dien-12-ol	0.4
1435	1438	Aromadendrene	0.6			Monoterpene hydrocarbons	31.7
1452	1454	α-Humulene	2.7			Oxygenated monoterpenoids	4.9
1471	1478	γ-Muurolene	0.9			Sesquiterpene hydrocarbons	24.3
1473	1476	γ-Gurjunene	1.4			Oxygenated sesquiterpenoids	27.9
1475	1482	α-Amorphene	0.3			Others	2.1
						Total identified	90.9

RI_(calc)_: Retention indices determined with respect to a homologous series of *n*-alkanes on a ZB-5ms column. RI_(db)_: Retention indices obtained from the databases [21,22,23].

**Table 5:** Twenty-four-hour mosquito larvicidal activities of Myrtaceae essential oils.

Essential Oil	LC_50_ (95% Fiducial Limits)	LC_90_ (95% Fiducial Limits)	χ^2^	*p*
	*Aedes aegypti*			
*Baeckea frutescens* leaf EO	23.00 (20.38–25.75)	40.05 (35.75–46.71)	6.512	0.039
*Callistemon citrinus* leaf EO	22.37 (18.62–25.88)	57.34 (50.00–69.06)	0.6655	0.717
*Callistemon citrinus* fruit EO	17.27 (15.30–19.03)	33.02 (29.82–38.04)	0.4348	0.805
*Melaleuca leucadendra* young leaf EO	nt	nt	---	---
*Melaleuca leucadendra* old leaf EO	7.400 (6.308–8.612)	18.29 (16.05–21.47)	30.77	0.000
*Melaleuca leucadendra* stem bark EO	17.14 (14.73–19.21)	36.25 (32.42–42.31)	2.244	0.326
*Melaleuca leucadendra* fruit EO	13.90 (11.03–16.02)	31.76 (28.40–37.25)	0.5750	0.750
*Melaleuca leucadendra* branch tip EO	21.99 (19.80–24.57)	37.63 (33.67–43.39)	2.277	0.517
*Syzygium nervosum* leaf EO	28.63 (24.83–32.87)	61.41 (53.99–72.38)	3.792	0.285
	*Aedes albopictus*			
*Baeckea frutescens* leaf EO	25.73 (23.68–28.39)	37.01 (33.33–43.13)	0.4209	0.810
*Callistemon citrinus* leaf EO	nt	nt	---	---
*Callistemon citrinus* fruit EO	nt	nt	---	---
*Melaleuca leucadendra* young leaf EO	nt	nt	---	---
*Melaleuca leucadendra* old leaf EO	nt	nt	---	---
*Melaleuca leucadendra* stem bark EO	19.31 (16.83–21.60)	40.91 (36.56–47.59)	0.5986	0.741
*Melaleuca leucadendra* fruit EO	19.17 (16.89–21.32)	39.08 (34.96–45.47)	4.7420	0.093
*Melaleuca leucadendra* branch tip EO	nt	nt	---	---
*Syzygium nervosum* leaf EO	nt	nt	---	---
	*Culex quinquefasciatus*			
*Baeckea frutescens* leaf EO	81.72 (76.16–87.75	112.7 (104.7–123.6)	3.097	0.078
*Callistemon citrinus* leaf EO	73.60 (64.87–85.83)	172.2 (135.9–249.1)	57.10	0.000
*Callistemon citrinus* fruit EO	17.30 (11.04–22.56)	77.42 (66.07–95.50)	63.93	0.000
*Melaleuca leucadendra* young leaf EO	46.62 (42.65–51.45)	70.10 (62.93–82.10)	0.2083	0.648
*Melaleuca leucadendra* old leaf EO	6.618 (3.635–9.183)	32.80 (27.99–40.13)	5.474	0.361
*Melaleuca leucadendra* stem bark EO	21.35 (13.62–28.02)	100.2 (84.4–126.2)	86.78	0.000
*Melaleuca leucadendra* fruit EO	26.20 (19.47–32.30)	91.81 (78.04–114.46)	46.32	0.000
*Melaleuca leucadendra* branch tip EO	43.69 (40.13–47.81)	64.43 (58.27–74.71)	0.02181	0.883
*Syzygium nervosum* leaf EO	46.09 (40.59–52.38)	95.07 (84.44–109.96)	1.061	0.786

LC_50_ and LC_90_ in μg/mL. nt = not tested.

**Table 6:** Forty-eight-hour mosquito larvicidal activities of Myrtaceae essential oils.

Essential Oil	LC_50_ (95% Confidence Limits)	LC_90_ (95% Confidence Limits)	χ^2^	*p*
	*Aedes aegypti*			
*Baeckea frutescens* leaf EO	15.31 (11.25–18.31)	34.69 (30.31–42.30)	2.418	0.298
*Callistemon citrinus* leaf EO	21.60 (17.74–25.13)	56.87 (49.55–68.64)	1.104	0.576
*Callistemon citrinus* fruit EO	16.80 (14.85–18.50)	31.91 (28.87–36.66)	0.2493	0.883
*Melaleuca leucadendra* young leaf EO	nt	nt	---	---
*Melaleuca leucadendra* old leaf EO	1.379 (1.127–1.626)	5.066 (4.173–6.551)	119.9	0.000
*Melaleuca leucadendra* stem bark EO	13.96 (10.91–16.21)	33.15 (29.54–39.08)	1.115	0.573
*Melaleuca leucadendra* fruit EO	9.071 (3.729–12.276)	30.90 (27.21–37.34)	1.180	0.554
*Melaleuca leucadendra* branch tip EO	15.79 (14.01–17.73)	28.64 (25.53–33.35)	2.103	0.551
*Syzygium nervosum* leaf EO	11.97 (5.54–16.89)	53.97 (45.87–67.18)	5.746	0.125
	*Aedes albopictus*			
*Baeckea frutescens* leaf EO	23.98 (21.76–26.57)	37.63 (33.75–43.80)	1.375	0.503
*Callistemon citrinus* leaf EO	nt	nt	---	---
*Callistemon citrinus* fruit EO	nt	nt	---	---
*Melaleuca leucadendra* young leaf EO	nt	nt	---	---
*Melaleuca leucadendra* old leaf EO	nt	nt	---	---
*Melaleuca leucadendra* stem bark EO	17.09 (14.89–19.01)	34.53 (31.02–40.08)	1.050	0.592
*Melaleuca leucadendra* fruit EO	17.34 (14.79–19.55)	37.85 (33.75–44.37)	3.9440	0.139
*Melaleuca leucadendra* branch tip EO	nt	nt	---	---
*Syzygium nervosum* leaf EO	nt	nt	---	---
	*Culex quinquefasciatus*			
*Baeckea frutescens* leaf EO	64.06 (56.83–72.12)	116.6 (103.4–137.2)	4.937	0.026
*Callistemon citrinus* leaf EO	49.18 (39.75–60.67)	227.8 (147.4–549.1)	16.79	0.000
*Callistemon citrinus* fruit EO	16.02 (12.54–19.77)	72.19 (60.64–91.68)	61.56	0.000
*Melaleuca leucadendra* young leaf EO	30.37 (21.56–36.81)	72.32 (63.07–88.25)	4.561	0.033
*Melaleuca leucadendra* old leaf EO	1.819 (1.262–2.394)	14.40 (11.04–20.43)	30.79	0.000
*Melaleuca leucadendra* stem bark EO	12.02 (5.71–16.91)	64.16 (55.04–78.56)	55.71	0.000
*Melaleuca leucadendra* fruit EO	17.38 (12.96–21.46)	88.42 (65.61–143.30)	17.23	0.000
*Melaleuca leucadendra* branch tip EO	23.78 (12.17–31.00)	66.12 (57.18–82.37)	2.383	0.123
*Syzygium nervosum* leaf EO	22.74 (16.64–28.33)	75.02 (64.50–91.30)	11.25	0.010

LC_50_ and LC_90_ in μg/mL. nt = not tested.

**Table 7:** Antimicrobial activities of Myrtaceae essential oils.

Sample	*Enterococcus faecalis*	*Staphylococcus aureus*	*Candida albicans*
	MIC (μg/mL)		
*Baeckea frutescens* leaf EO	64	nt	16
*Callistemon citrinus* leaf EO	32	256	16
*Callistemon citrinus* fruit EO	16	nt	128
*Melaleuca leucadendra* old leaf EO	32	64	128
*Melaleuca leucadendra* stem bark EO	16	64	64
*Melaleuca leucadendra* fruit EO	32	64	256
*Syzygium nervosum* leaf EO	32	nt	128
Streptomycin	256	256	nt
Nistatin	nt	nt	8
	IC_50_ (μg/mL)		
*Baeckea frutescens* leaf EO	33.56	nt	8.67
*Callistemon citrinus* leaf EO	16.67	128.00	8.67
*Callistemon citrinus* fruit EO	8.89	nt	32.67
*Melaleuca leucadendra* old leaf EO	16.72	33.23	65.56
*Melaleuca leucadendra* stem bark EO	8.32	32.23	34.22
*Melaleuca leucadendra* fruit EO	15.98	32.89	128.35
*Syzygium nervosum* leaf EO	17.00	nt	65.33

MIC = minimum inhibitory concentration, EO = essential oil, nt = not tested, IC_50_ = median inhibitory concentration.

**Table 8:** Antimicrobial activities (MIC, μg/mL) of essential oil components from the literature.

Compound	*Enterococcus faecalis* [Ref]	*Staphylococcus aureus* [Ref]	*Candida albicans* [Ref]
α-pinene	8000 [62] >4000 [63] inactive [64]	13.6 [65] 45.7 [66] 312 [60] 800 [62] 1600 [67] 1300–2500 [68] >32 [59]	12 [59] 156 [60] 800 [67] >1000 [69]
β-pinene	60 [70] 2500 [71] >4000 [63]	3.0 [59] 41.3 [66] 600 [70] 1600 [67] >20 [65]	1.0 [59] 60 [70] 100 [69] 1600 [67]
*p*-cymene	600 [72] inactive [73]	2000 [67] >32 [59] >10,000 [68] >80,000 [74]	100 [69] 1600 [67] >32 [59] >80,000 [61]
limonene	27,000 [75]	24 [59] 32.1 [66] 312 [60] >20 [65] >10,000 [68]	10 [59] 1000 [69] 1250 [60]
1,8-cineole	7500 [64] 23,000 [75] >8000 [76] inactive [62]	32 [59] 625 [60] 5000 [74] >10,000 [68]	312 [60] 10,000 [74] 40,000 [61] >32 [59] >1000 [69]
γ-terpinene	no data	>32 [59] >80,000 [74]	6.0 [59] 100 [69] >80,000 [61]
α-terpineol	>1000 [77]	1250 [60] 2500 [74] >20 [65]	1200 [61] 1250 [60] 2500 [74]
(*E*)-caryophyllene	6 [78] 60 [70] 2500 [71] >4000 [63] inactive [79]	5.1 [65] 30.3 [66] 60 [78] 312 [60] 9100 [79] >10,000 [68]	1250 [60] >1000 [69] inactive [78] inactive [79]
α-humulene	6 [70] >400 [80]	2.6 [65] 312 [60] >10,000 [68] inactive [70]	625 [60] inactive [70]

**Table 9:** Collection details and essential oil yields of four species of Myrtaceae from central Vietnam.

Species	Vietnamese Name	Collection Site	Voucher Number	Part	% Yield
*Baeckea frutescens* L.	Chổi xể, Chổi trện, Chóp máu, Thanh hao, Thanh liễu	Hoa Vang district, Da Nang city (16°1′10.1″ N, 108°06′01.3″ E, elev. 27 m), in January 2019.	NHH7	Leaf	2.23
*Melaleuca leucadendra* (L.) L.	Tràm lá dài, tràm lá hẹp	Hoa Vang district, Da Nang city (16°1′10.1″ N, 108°06′01.3″ E, elev. 27 m), in February 2019.	NHH4	Young leaf	1.22
				Old leaf	1.43
				Stem bark	0.91
				Fruit	1.12
				Branch tip	1.10
*Callistemon citrinus* (Curtis) Skeels	Tràm bông đỏ, Tràm liễu, Kiều nhụy, Kiều hùng	Garden for Medicinal Plant Conservation, Duy Tan University, Hoa Khanh district, Da Nang city (16°02′57.6″ N, 108°09′34.5″ E, elev 8 m), in November 2018.	NHH6	Leaf	0.62
				Fruit	0.34
*Syzygium nervosum* DC.	Vối, Trâm vối, Trâm nắp	Garden for Medicinal Plant Conservation, Duy Tan University, Hoa Khanh district, Da Nang city (16°02′57.6″ N, 108°09′34.5″ E, elev. 8 m), in January 2019.	NHH10	Leaf	0.20
